# Dissection of lymph node on longevity in breast and colorectal operation

**DOI:** 10.6026/973206300223636

**Published:** 2026-06-30

**Authors:** Deepak Premnarayan Gupta1, Ritesh Yadav, Priyanshu Jain, Talha Saad, Akash Wamanrao Karale, Satyendra Mishra, Roopa Agrawal

**Affiliations:** 1 Department of Anaesthesia, Bundelkhand Government Medical College, Sagar, Madhya Pradesh, India; 2 Department of General Medicine, SRVS Medical College, Shivpuri, Madhya Pradesh, India; 3 Department of Emergency Medicine, Bundelkhand Medical College Sagar, Madhya Pradesh, India; 4 Department of Respiratory Medicine, Bundelkhand Medical College Sagar, Madhya Pradesh, India; 5 Department of surgery, Bundelkhand Medical College, Sagar, Madhya Pradesh, India; 6 Department of Paediatrics, Bundelkhand Government Medical College, Sagar, Madhya Pradesh, India

**Keywords:** Breast cancer, colorectal carcinoma, lymph node removal, cancer surgery, survival outcomes

## Abstract

The dissection of the lymph nodes is customarily accomplished but argumentation pursues for the extent requisition to acquire lessen complications after the operation and diminished cancer burden. Therefore, it is of interest to appraise how lymph node excision impacts challenges after the surgery and influences the survival in respondents' encountered operation for the management of breast and colorectal cancer. The identification of metastatic nodes was substantially heightened with sufficient removal in juxtapose to those with insufficient removal (52.4% vs. 29.7%; p < 0.01), emphasizing the purpose of sufficient node harvesting. Thus, data shows a judicious and selective method of lymph node removal that guarantees precise cancer staging and survival advantages while reducing superfluous surgical morbidity.

## Background:

Breast and colorectal malignancies rank among the most common cancers globally, contributing substantially to cancer-related morbidity and mortality. While improvements in early diagnosis, combined therapeutic approaches and rigorous post-operative surveillance have enhanced survival rates, surgical removal remains the fundamental curative intervention for both diseases [1]. The excision and examination of regional lymph nodes constitute a vital component of cancer surgery, playing a crucial role in accurate disease classification, guiding decisions regarding supplementary treatment and predicting long-term prognosis. The detection of lymph node metastasis indicates tumour aggressiveness and potential for widespread dissemination. In breast cancer, axillary lymph node metastasis directly influences prognosis, determines the necessity for chemotherapy or radiotherapy and informs disease staging [2, 3]. In carcinoma of colorectal region, the lymph nodes hold powerful probable of reiteration and overall output resulting in consideration of removal of lymph nodes as a vital key element of carcinoma operation. Nonetheless, excessive removal of lymph nodes is able to heightened control locally with lesser reiteration as discovered by surgeons but it may result in substantial challenges [4]. Axillary lymph node removal is able to cause pain, stiff shoulder, loss of sensation and lymphedema whilst carcinoma of colorectal region results in lengthier duration of surgery, greater loss of blood and injured nerves and blood vessels. It impacts the recovery after the surgery ultimately reducing the qualitative life [5, 6]. Therefore, it is of interest to outline how the area of lymph node removal impacting the morbidity after surgery and survival outputs in respondents scheduled for operation for carcinoma of breast and colorectal region.

## Materials and Methods:

## Research design and sample: 

The current survey was accomplished prospectively which is cross-sectional and observational in type, executed for the span of 2 years from April 2023 till March 2025. The verified surgical 186 respondents for the management of breast and colorectal cancer were registered. The respondents incorporated were adults with 18 years of age and above enduring breast and colorectal cancer (histopathological verified) which falls amid stage I-III clinically and has been outlined for conservative removal by operation. Exclusions incorporated were the cases of recurrence, receiving neoadjuvant treatment previously, morbid conditions in which operation is impeded or proved cases of metastasis. The size of the respondents computed by utilizing the aforementioned formulation,

n = (Z_α/2_ + Zβ)^2^ x [P_1_ (1-P_1_) + P_2_ (1-P_2_)])/ (P_1_-P_2_)^2^

Z(_α/2_)=1.96, Zβ=0.84, P_1_=0.85, P_2_=0.60

Total (minimum) = 92 respondents

We add up 10% attrition ≈ 101 respondents To boost the statistical power and validating subgroup survey by the variety of carcinoma, the size of the respondents was inflated to 186 respondents.

## Operative approach:

[1] Carcinoma of breast: The respondents were managed either by breast conservative operation or radical removal of breast tissue conservatively. Sentinel lymph node biopsy was accomplished in clinically verified node-negative patients. The removal of axillary lymph nodes was accomplished.

[2] Carcinoma of colorectal: The respondents encountered carcinoma removal attuned to the its region incorporating right or left half resection of colon, resection of sigmoid or anterior region alongside removal of mesenteric lymph nodes with standardization.

The compilation of data either clinical or surgical incorporates demography of respondents like age and gender, staging of carcinoma with its histological features. The variety of operation executed. Complications arising after surgery, the orchestration of adjuvant therapy, recurrence of the condition and rate of survival and positive as well as total lymph node count were tabulated and followed up for 2 years span. The primary outputs were delineating the disease-free survival (DFS) and overall survival (OS). The secondary outputs incorporate morbid conditions after surgery and inpatient duration.

## Ethical considerations:

All the respondents furnish signed informed consent. Ethical clearance was acquired by the ethical committee. This research follows the Helsinki's declaration. The data of the respondents were kept private and safe.

## Statistical analysis:

The data were analysed utilizing the SPSS software. The outputs of survival were computed by employing multivariate regression and Kaplan-Meier analysis disparity amid groups were analysed by employing long-rank test. The value of P <0.05 depicts the statistical disparity.

## Results:

Colorectal cancer patients had a substantially higher average number of lymph nodes recovered (17.6 ± 5.4) than breast cancer patients (14.1 ± 4.8) (p=0.001). However, there was no significant difference in the proportion of patients with nodal positive between the two cancer groups (41.3 percent vs. 47.6%, p=0.37). Similarly, nodal positivity rates within the adequate and inadequate lymph node dissection groups were comparable, indicating that cancer type did not significantly influence nodal detection rates as seen in Table 1. Patients who received extensive lymph node dissection had significantly higher postoperative morbidity than those who underwent sufficient dissection. The total complication rate was higher in the extensive dissection group (34.1% vs. 21.3%, p=0.03). Seroma development was substantially more common after extensive dissection (14.6% vs. 6.5%, p=0.04), while lymphedema exhibited a borderline significant increase (9.7% vs. 4.9%, p=0.05). Excessive lymph node dissection led to a considerably longer hospital stay (9.1 ± 2.3 vs. 6.7 ± 1.9 days, p < 0.001). There was no significant difference in wound infection rates between the groups (p = 0.29).

## Discussion:

To obtain the precise outputs after the surgery, sufficient removal of the lymph nodes is vital in carcinoma of breast and colorectal cases as discussed in this current research survey. Precise assessment of the lymph nodes entitles the beginning of adjuvant treatment in timely manner, diminishing the likelihood of reiteration and better survival. In the literature it was noted that huge excision of lymph node furnishes better survival in the treatment of carcinoma as discovered by Fisher *et al.* [7] in the present research, it is tied to heightened morbidity after surgery, affecting the qualitative life of the respondents. These observations are similar with the research executed by Giuliano *et al.* who uncovered that conservative removal of axillary lymph nodes in carcinoma of breast cases did not affect the DFS and OS if staging of lymph nodes was correctly obtained [8]. Galimberti *et al.* uncovered the similar observations as discovered in the present research [9]. Swanson *et al.* demonstrated that examining a minimum of 12 lymph nodes was associated with improved survival, largely because it allows more accurate pathological staging and helps guide the appropriate use of adjuvant chemotherapy [10]. In a similar vein, Chen and Bilchik reported that patients with inadequate nodal retrieval experienced higher rates of recurrence and poorer long-term outcomes [11]. The findings of our study reinforce these observations, particularly in relation to stage migration. When nodal sampling is insufficient, there is a greater risk of under staging, which may lead to suboptimal treatment decisions and, consequently, a less favourable prognosis [12]. Supporting our results, Le Voyer *et al.* observed that removing lymph nodes beyond recommended thresholds did not yield proportional survival benefits [13]. These findings suggest that overly aggressive lymphadenectomy may place patients at unnecessary risk without offering meaningful oncologic advantage. Taken together, the available evidence indicates that lymphadenectomy should be regarded as a biologically informed and carefully tailored intervention rather than a purely mechanical or volume-driven procedure. Adequate nodal clearance contributes to improved disease-free and overall survival by enabling precise staging and appropriate allocation of adjuvant therapy [14]. Conversely, excessively aggressive dissection may increase morbidity while providing limited additional benefit [15]. Nonetheless, the single-center design and relatively short follow-up period require that the results be interpreted with caution. Future multicenter studies with extended follow-up are necessary to better define optimal nodal harvest thresholds across different tumor types and stages. Incorporating patient-reported outcomes will also help clarify the long-term functional and quality-of-life implications. Furthermore, advances in minimally invasive and robotic-assisted techniques may offer opportunities to achieve precise nodal clearance while minimizing post-operative morbidity.

## Conclusion:

The sufficient removal of the lymph nodes is the vital approach for precise staging and greater survival in carcinoma of breast and colorectal region. The huge removal of lymph nodes heightens the morbidity after surgery with no substantial benefit in terms of survival. Attuned approach of the removal of lymph nodes regarding the staging of cancer and its biology precise the results and conserves the function and qualitative life after the surgery.

## Figures and Tables

**Table 1:** Removal of lymph node its related pathological observations, morbidity after surgery, survival outputs and multivariate regression analysis

**Lymph Node Yield and Nodal Positivity**
**Parameter**	**Breast Cancer**	**Colorectal Cancer**	**p-value**
Mean lymph nodes retrieved	14.1 ± 4.8	17.6 ± 5.4	0.001
Nodal positivity	43 (41.3%)	39 (47.6%)	0.37
Positivity in adequate group	52.40%	55.10%	0.41
Positivity in inadequate group	29.70%	31.40%	0.62
**Postoperative Morbidity and Hospital Stay**
**Parameter**	**Adequate LND (n=122)**	**Excessive LND (n=41)**	**p-value**
Overall complications	26 (21.3%)	14 (34.1%)	0.03
Seroma	8 (6.5%)	6 (14.6%)	0.04
Wound infection	10 (8.1%)	5 (12.2%)	0.29
Lymphedema	6 (4.9%)	4 (9.7%)	0.05
Mean inpatient duration (days)	6.7 ± 1.9	9.1 ± 2.3<0.001
**Survival Outcomes at 24 Months**
**Outcome**	**Adequate LND**	**Inadequate LND**	**p-value**
Disease recurrence	20 (16.4%)	21 (31.3%)	0.01
Disease-free survival	83.60%	68.70%	0.004
Overall survival	88.00%	72.10%	0.002
**Multivariate Cox Regression Analysis**
**Variable**	**Hazard Ratio (HR)**	**95% CI**	**p-value**
Inadequate nodal retrieval	1.94	1.18 - 3.19	0.009
Nodal positivity	2.73	1.64 - 4.53	<0.001
Stage III disease	2.48	1.41 - 4.37	0.002
